# Case Report: Event-Related Desynchronization Observed During Volitional Swallow by Electroencephalography Recordings in ALS Patients With Dysphagia

**DOI:** 10.3389/fnbeh.2022.798375

**Published:** 2022-02-16

**Authors:** Akari Ogawa, Satoko Koganemaru, Toshimitsu Takahashi, Yuu Takemura, Hiroshi Irisawa, Masao Matsuhashi, Tatsuya Mima, Takashi Mizushima, Kenji Kansaku

**Affiliations:** ^1^Cognitive Motor Neuroscience, Human Health Sciences, Graduate School of Medicine, Kyoto University, Kyoto, Japan; ^2^Department of Regenerative Systems Neuroscience, Human Brain Research Center, Graduate School of Medicine, Kyoto University, Kyoto, Japan; ^3^Department of Physiology, Dokkyo Medical University, Shimotsuga-gun, Tochigi, Japan; ^4^Department of Rehabilitation Medicine, Dokkyo Medical University, Shimotsuga-gun, Tochigi, Japan; ^5^Department of Epilepsy, Movement Disorders and Physiology, Graduate School of Medicine, Kyoto University, Kyoto, Japan; ^6^The Graduate School of Core Ethics and Frontier Sciences, Ritsumeikan University, Kyoto, Japan

**Keywords:** event-related desynchronization, amyotrophic lateral sclerosis, dysphagia, electroencephalography, cerebral cortex

## Abstract

Dysphagia is a severe disability affecting daily life in patients with amyotrophic lateral sclerosis (ALS). It is caused by degeneration of both the bulbar motor neurons and cortical motoneurons projecting to the oropharyngeal areas. A previous report showed decreased event-related desynchronization (ERD) in the medial sensorimotor areas in ALS dysphagic patients. In the process of degeneration, brain reorganization may also be induced in other areas than the sensorimotor cortices. Furthermore, ALS patients with dysphagia often show a longer duration of swallowing. However, there have been no reports on brain activity in other cortical areas and the time course of brain activity during prolonged swallowing in these patients. In this case report, we investigated the distribution and the time course of ERD and corticomuscular coherence (CMC) in the beta (15–25 Hz) frequency band during volitional swallow using electroencephalography (EEG) in two patients with ALS. Case 1 (a 71-year-old man) was diagnosed 2 years before the evaluation. His first symptom was muscle weakness in the right hand; 5 months later, dysphagia developed and exacerbated. Since his dietary intake decreased, he was given an implantable venous access port. Case 2 (a 64-year-old woman) was diagnosed 1 year before the evaluation. Her first symptom was open-nasal voice and dysarthria; 3 months later, dysphagia developed and exacerbated. She was given a percutaneous endoscopic gastrostomy. EEG recordings were performed during volitional swallowing, and the ERD was calculated. The average swallow durations were 7.6 ± 3.0 s in Case 1 and 8.3 ± 2.9 s in Case 2. The significant ERD was localized in the prefrontal and premotor areas and lasted from a few seconds after the initiation of swallowing to the end in Case 1. The ERD was localized in the lateral sensorimotor areas only at the initiation of swallowing in Case 2. CMC was not observed in either case. These results suggest that compensatory processes for cortical motor outputs might depend on individual patients and that a new therapeutic approach using ERD should be developed according to the individuality of ALS patients with dysphagia.

## Introduction

Amyotrophic lateral sclerosis (ALS) is a degenerative disorder affecting the upper and lower motor neurons (Brooks, 1996). Dysphagia is a disability caused by oropharyngeal muscle weakness during disease progression; most ALS patients encounter this problem, which severely affects their activities of daily living (ADL) and quality of life (QOL). In general, damage to the upper and lower motoneurons results in dynamic cortical reorganization in multiple regions (Elbert and Rockstroh, 2004; Jurkiewicz et al., 2007; Fortanier et al., 2019). However, there have been few studies on cortical reorganization during swallowing in ALS patients with dysphagia (Li et al., 2009a; Teismann et al., 2011).

In previous studies using PET and functional MRI (fMRI), cortical activation was widely observed in the primary motor (M1), somatosensory, supplementary, and pre-motor cortices during volitional swallowing in healthy subjects (Ertekin and Aydogdu, 2003; Michou and Hamdy, 2009). ALS patients with dysphagia showed less cortical activity in the bilateral sensorimotor areas during volitional self-paced swallowing, compared with healthy subjects in a previous magnetoencephalography (MEG) study using event-related desynchronization (ERD) (Teismann et al., 2011). ERD is a decrease in power spectral density in the alpha (10–12 Hz) and beta frequency bands (15–25 Hz) observed during voluntary movements and is thought to reflect motor-related cortical activity (Pfurtscheller and Lopes Da Silva, 1999; Li et al., 2018; Chen et al., 2020; Spadone et al., 2020; Xie et al., 2021). The ERDs within the beta frequency band (13–30 Hz) in bilateral sensorimotor areas were less prominent in patients with ALS, and the lower the ERD, the more severe the dysphagia they had (Teismann et al., 2011), suggesting that the reduced ERD reflected the disturbed function of the degenerated cortico-motoneurons. Although the previous report showed lower activities in the sensorimotor areas (Teismann et al., 2011), there have been no report of the whole brain areas during swallow. In general, corticomotoneuronal lesions reorganize the wide-spread brain areas probably for compensation of the motor function (Ward et al., 2004; Ward, 2005). Therefore, other brain areas than sensorimotor areas would be recruited during swallowing in patients with dysphagia. In addition, swallow durations are often prolonged due to disturbances in the oral and pharyngeal phases in ALS patients (Ertekin et al., 2000; Teismann et al., 2011; Jani and Gore, 2016). However, there have been no reports about the time course of ERDs during prolonged swallowing in patients with ALS.

Recently, we have succeeded in recording ERDs within beta frequency band during swallowing in healthy subjects using electroencephalography (EEG), which is a convenient method for evaluating cortical activity in clinical practice (Koganemaru et al., 2021). Therefore, we investigated the time course of the ERD within beta band frequency in the whole brain areas in the prolonged swallow duration in two ALS patients using EEG.

Corticomuscular coherence (CMC) has also been reported in EEG studies using motor tasks of the upper and lower limbs (Mima and Hallett, 1999a). CMC is supposed to indicate corticomotoneuronal activation (Mima and Hallett, 1999a). Since predominant CMC was observed in the bilateral sensorimotor, premotor, and inferior prefrontal areas during volitional swallowing in healthy volunteers in our preliminary EEG study (Koganemaru et al., 2021), we also investigated CMC during swallowing in ALS patients.

We hypothesized that the ERD during the swallow could be found in other cortical areas than the medial sensorimotor areas, it would be changed within a prolonged swallow period and CMC would be reduced due to cortico-bulbar degeneration in the dysphagic ALS patients. There has been no previous report showing ERD in the multiple cortical areas, the time course of ERD and the CMC in ALS patients. Therefore, we investigated them in the two ALS patients.

## Case Description

### Cases

Case 1 was a 71-year-old right-handed man. He became aware of muscle weakness in his right hand at the age of 69 years. He was diagnosed with ALS at the age of 70 years. At the age of 71 years, dietary intake decreased due to exacerbation of dysphagia (Table 1, in detail). He was admitted to the hospital for the construction of an implantable venous access port for nutrition intake. He could take water without thickening, jelly, and enzyme-degraded food by adjusting it to a mouthful volume. The % vital capacity (%VC) was maintained (%VC, 84.8%) and the forced expiratory volume was decreased (FEV1.0%, 52.2%). The patient's ADL score was independent. In the video-fluorography (VF) evaluation, pharyngeal phase difficulty was found.

**Table 1:** Clinical findings of Case 1 and Case 2.

**Years**	**Clinical findings**
**Case 1**	
0 (Onset)	Aware of right-hand weakness
0.4	Developed open-nasal voice and hoarseness
1.1	Showed swallow difficulty
	• Diagnosed with ALS according to the following findings: • Upper motor neuron deficits: • Exaggerated jaw jerk and abnormal sucking reflex • Exaggerated deep tendon reflexes of right biceps and triceps brachii muscles and left patella • Lower motor neuron deficits: • Fasciculation of tongue, bilateral forearm and lower leg muscles • Weakness of bilateral deltoid, right biceps and triceps brachii, right finger flexor and extensor, bilateral iliopsoas, hamstrings and quadriceps muscles • Atrophy of tongue, vocal cord, bilateral thenar and right first dorsal interossei muscles • Hoarseness due to vocal cord atrophy and swallowing difficulty • Needle EMG findings: fibrillation, positive sharp wave, fasciculation, large or polyphasic motor unit potentials and reduced recruitments in the right trapezius, biceps brachii, extensor digitorum, erector spinae (the level of Th10) and tibialis anterior muscles • No sensory disturbances nor ataxia • Normal laboratory data (blood and cerebrospinal fluid test), brain and spinal MR images without explainable findings for the symptoms
	Edaravone administered
1.6	Admitted to the hospital to construct totally implantable central venous access port ALSFRS-R 36 MWST: 3a, RSST: 1 Evaluated EEG
**Case 2**	
0 (Onset)	Developed open-nasal voice and dysarthria
0.5	• Diagnosed with ALS according to the following findings: • Upper motor neuron deficits: • Exaggerated deep tendon reflexes of bilateral biceps brachii muscles and patellae • Lower motor neuron deficits: • Fasciculation of tongue and inferior eyelids • Weakness of neck flexor, bilateral deltoid, biceps and triceps brachii, and extensor digitorum muscles • Atrophy of tongue • Facial nerve palsy • Needle EMG findings: fibrillation, positive sharp wave, fasciculation, and reduced interference and recruitments in the right trapezius, biceps brachii, extensor digitorum, erector spinae (the level of Th10) and tibialis anterior muscles • Dysarthria • No sensory disturbances nor ataxia • Normal laboratory data (blood test and cerebrospinal fluid test), normal brain and spinal MR images
	Edaravone administered (60 mg/day for 14 days)
1	Starting to use the bilevel positive airway pressure
1.5	• Admitted to constructed PEG • Evaluated EEG • ALSFRS-R 37 • MWST: 4, RSST: 2

*ALSFRS-R, ALS functional rating scale-revised (Cedarbaum et al., 1999); MWST, modified water swallowing test; RSST, repetitive saliva swallowing test*.

Case 2 was a 64-year-old right-handed woman. She developed an open-nasal voice and dysarthria and was diagnosed with ALS at the age of 63 years (Table 1, in detail). The patient was admitted to the hospital to construct percutaneous endoscopic gastrostomy (PEG) due to exacerbated dysphagia. On admission, she showed tongue atrophy, limited elevation, and inversion of the tongue. She could consume pasted food due to impaired mastication and dysphagia. The oral phase was more disturbed by the weak movements of the mandible and tongue in the VF. The %VC decreased (73.9%) and the FEV1.0% was maintained (88.0%). The patient's ADL score was independent.

### EEG and EMG Recordings

The patients were comfortably seated in an armchair during recording. The EEG signals were recorded using 32 electrodes. EEG electrodes were eego^TM^ sports active electrodes (ANT Neuro, Netherlands) and attached inside the EEG cap according to the 10–20 international electrode system. The EEG signals were amplified using an eego^TM^ sports amplifier. A CPz electrode was selected as the reference electrode. The impedance of all the electrodes was <15 kΩ. Data were recorded and saved at a sampling rate of 1 kHz. We concurrently recorded surface EMG using two pairs of bipolar silver electrodes attached to bilateral submental group muscles and the left orbicularis oris muscle according to previous studies (Okitsu et al., 1998; Nederkoorn et al., 1999; Ding et al., 2002; Vaiman, 2007). The electrodes were connected to a bipolar eego^TM^ sports amplifier (ANT Neuro, Netherlands). Swallowing movements were recorded with a triple-axis accelerometer placed on the anterior part of the participant's neck, similar to previous studies (Suntrup et al., 2014, 2015; Jestrovic et al., 2018; Suntrup-Krueger et al., 2018). Head movements during volitional swallowing were monitored using two cameras. An experimenter injected 2 mL of water into the oral cavity via a flexible tube with 3.3-mm diameter connected to the syringe before each swallow during the experiment. The patients were asked to perform volitional swallowing without moving their heads at their own pace with a waiting time of >3 s after the water infusion according to the previous report (Koganemaru et al., 2021). The tip of the tube was placed on the right of the mouth between the buccal part of the teeth and cheek, and fixed on the skin with tape, similar to the previous MEG studies (Suntrup et al., 2014, 2015; Suntrup-Krueger et al., 2018). They repeated the volitional swallow for 1 h to evaluate 100–150 times of voluntary swallow with 15–20 s of inter-intervals.

### Data Analysis

#### Pre-processing

**Figure 1:**
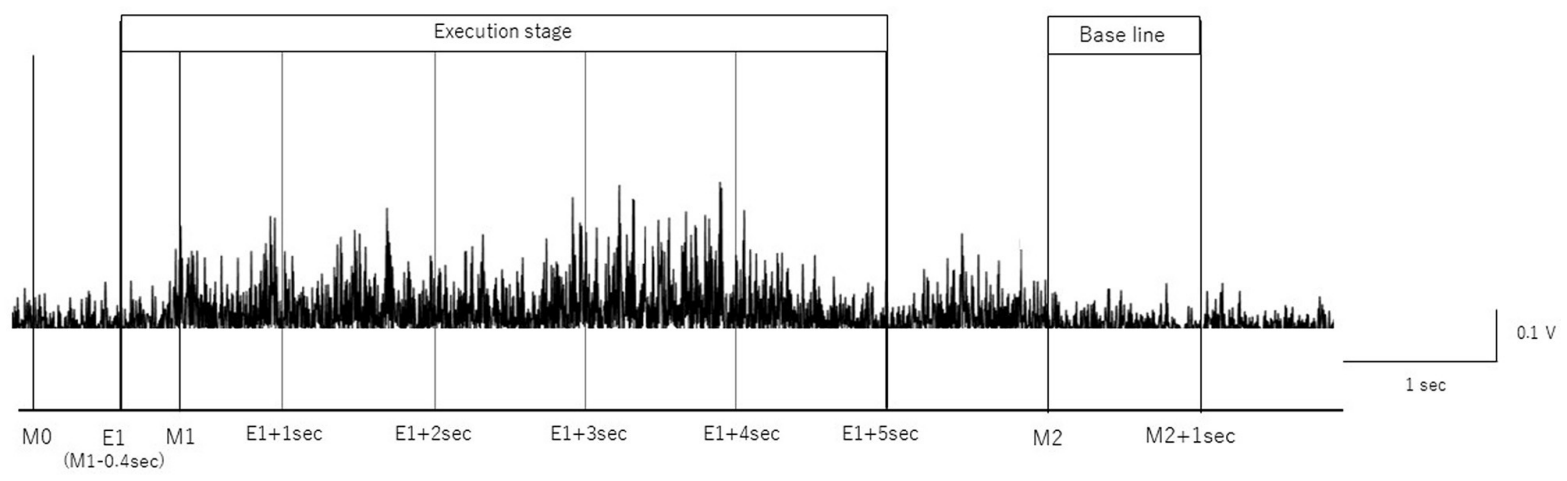
Definition of onset and offset of swallow. The onset and offset of volitional swallowing are defined according to swallow-related submental group muscle activities form Case 1. M0 is the E1 is the time to initiate swallowing, M1 is the time to start the main muscle activation, and M2 is the time to return to baseline. The surface EMG of the submental group muscle trace during a single swallow is shown.

We removed artifacts of the blink, electrooculographic activities, and muscle activities related with swallow movements from the EEG signals using independent component analysis (ICA) (Hyvärinen and Oja, 2000) using the EEGLAB MATLAB toolbox (Math Works Inc., USA) (Delorme and Makeig, 2004). We segmented EEG signals based on the onset and offset of each swallow, as determined by rectified EMG signals from submental group muscles according to previous MEG studies (Teismann et al., 2011; Suntrup et al., 2014, 2015; Suntrup-Krueger et al., 2018). The beginning of the main muscle activation (M1) was defined as the time point at which the amplitude of the rectified EMG signal continuously increased by more than 100% (Figure 1). Moreover, the onset of the first visible EMG activity of the swallowing movement (M0) was manually set to determine the total swallow duration (from M0 to M2). The end of the swallow specific muscle activity (M2) was defined as the time point when the amplitude of the rectified EMG signal continuously decreased by more than 50%, as in previous studies (Suntrup et al., 2014, 2015; Suntrup-Krueger et al., 2018). To analyze the event-related EEG signals, the onset of swallowing (E1) was defined as (M1–0.4 s) and the offset of the swallowing as M2, according to previous studies (Dziewas et al., 2009; Teismann et al., 2011; Suntrup et al., 2015). The time intervals are defined as follows:

(1) Execution stage: −0.4 to 4.6 s divided by 1 s duration in reference to M1 (from E1 to E1+ 5 s)
(2) Resting-stage: 0–1 s in reference to M2.

#### EEG Analysis

##### Event-Related Desynchronization

We calculated the power spectral density of the segmented EEG denoised by ICA using fast Fourier transform (FFT). We applied the FFT to 1,000 ms segments spanning the activation stage (from E1 to E1 + 5 s, divided into 1,000 ms intervals). The baseline for ERD analysis was 1,000 ms segments spanning the resting stage (from M2 s to M2 + 1.0 s). The upper and lower limits of the FFT were 500 and 1 Hz, respectively. The evaluated frequency ranged from 15 to 25 Hz. The ERD was calculated by taking the logarithm of the averaged power spectrum and subtracting that in the baseline from that in the activation stage. We obtained the average ERD of all channels in each patient in the beta (15–25 Hz) frequency. The 95% confidence limit for the EEG power was calculated for the number of trials (n) in each patient using the following equation (Halliday et al., 1995).


$$\text{Confidence}\;\text{limit}\;\left(95\%\right)=\pm \left(0.851\right)*{\left(\text{n}\right)}^{-\;1/2}$$

##### Corticomusclar Coherence

By using FFT, we computed the cross- and auto-spectra in the frequency domain of the EEG signals and rectified EMG signals from submental group muscles in the activation phase. The FFT was applied to 1,000 ms segments spanning from E1 to E1 + 5 s in the same way as with the calculation of ERD. The coherence is defined as cross-spectra normalized by auto-spectra, and it expressed by the following equation, in which *f*_*xx*_(*j*), *f*_y*y*_(*j*), and |*f*_x*y*_(*j*)| denote auto-spectra and cross-spectra at frequency *j* (Mima and Hallett, 1999a).


$$|{\text{R}}_{\text{xy}}\left(\text{j}\right){|}^{2}=\frac{|{\text{f}}_{\text{xy}}\left(\text{j}\right){|}^{2}}{{\text{f}}_{\text{xx}}(\text{j}){\text{f}}_{\text{yy}}(\text{j})}$$

The 95% confidence limit was calculated for the number of trials (n) in each patient using the following equation (Mima and Hallett, 1999a).


$$\text{Confidence}\;\text{limit}\;\left(95\%\right)=\;1-{\left(0.05\right)}^{1/(\text{n}-1)}$$

## Assessment

**Figure 2:**
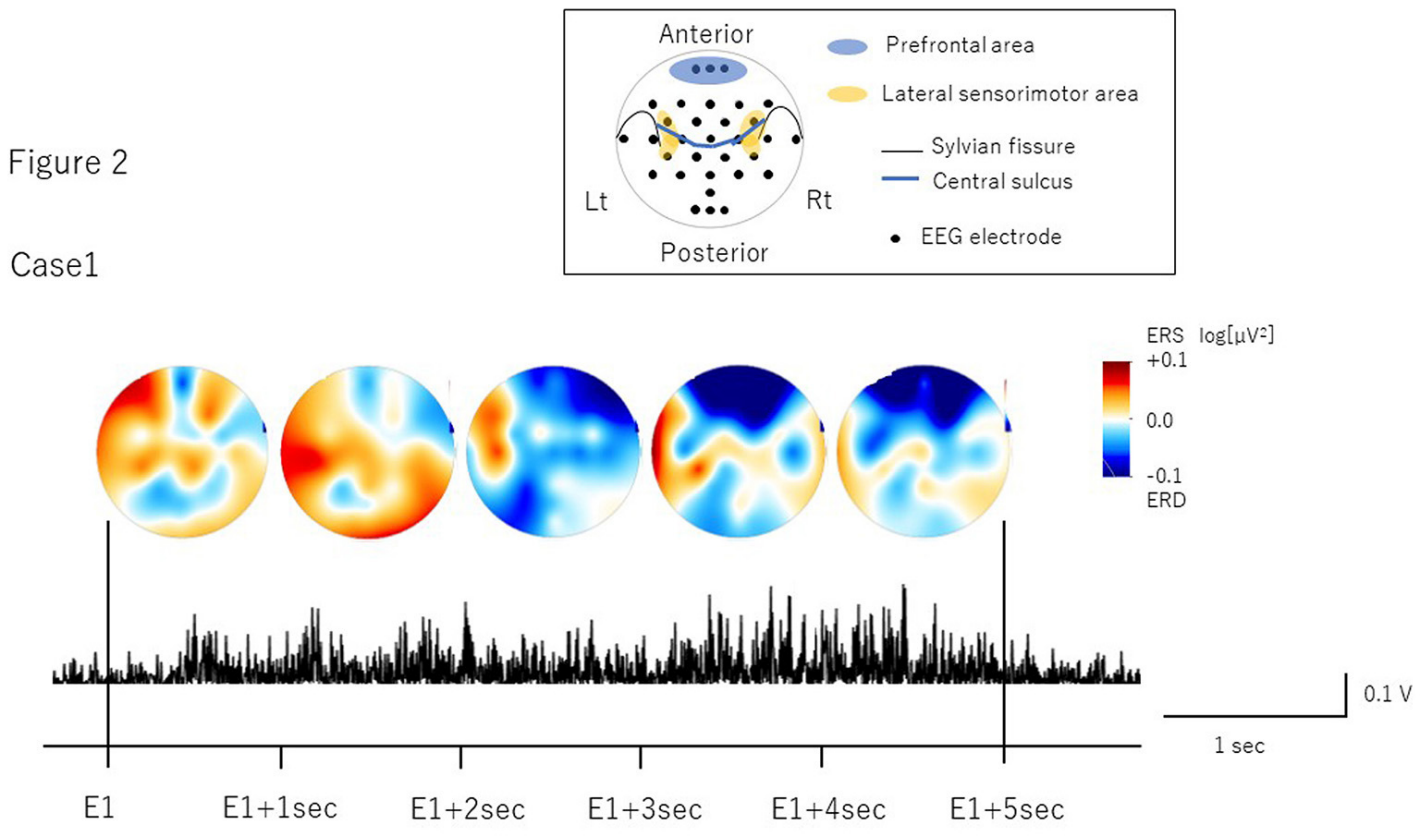
The topographic mapping of the averaged ERD/ERS and the EMG of submental group muscles in Case 1. The topographic mapping display of the swallow-related ERD or ERS every 1 s and the EMG of submental group muscles during a single swallow in Case 1 are shown. ERD/S are color-coded on the topomap display. ERD is prominent in the bilateral prefrontal areas from 3 s after the onset of swallowing to the end of swallowing.

**Figure 3:**
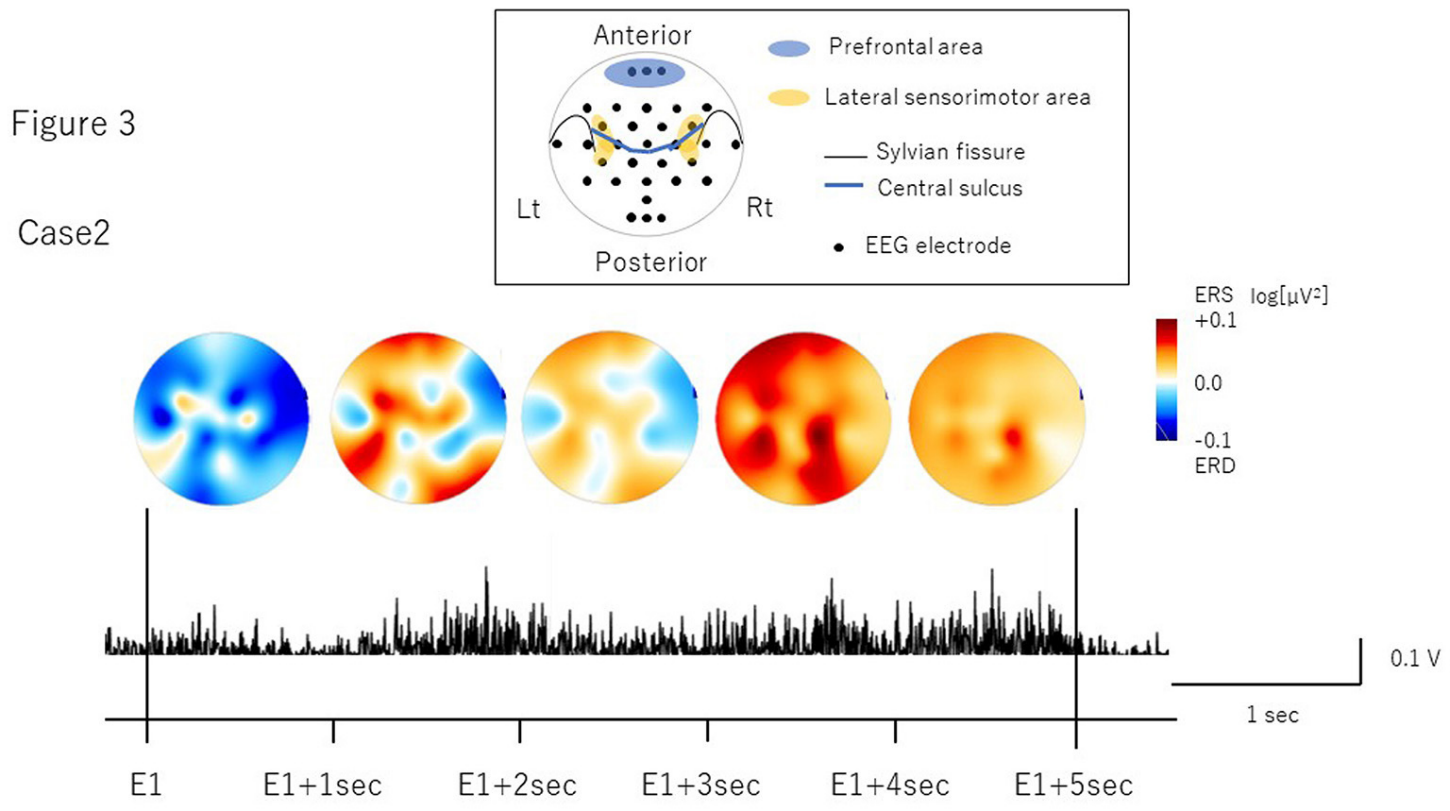
The topographic mapping of the averaged ERD/ERS and the EMG of submental group muscles in Case 2. The topographic mapping display of the swallow-related ERD or ERS every 1 s and the EMG of submental group muscles during a single swallow in Case 2 are shown. ERD/S are color-coded on the topomap display. ERD is prominent in the right lateral sensorimotor areas at the beginning of swallowing.

The number of volitional swallows in each patient was 120 times in Case 1 and 89 times in Case 2 during EEG recording. The averages of each swallow duration were 7.6 ± 3.0 s in Case 1 and 8.3 ± 2.9 s in Case 2. We visually inspected head movements monitored by cameras and found no head movements during swallowing in either case. During the recordings including the volitional swallow task, no adverse events occurred. During the volitional swallowing task, no aspiration occurred and the patients did not execute the double or more swallowing and did not swallow the saliva. We removed ~5% of the trials with <5 s of swallow duration so that we could investigate brain activity during prolonged swallow movements. Figures 2, 3 show the EMG of submental group muscles and the topographic mapping of the averaged ERD or event-related synchronization (ERS) every 1 s. The 95% confidence limit for the ERD were ± 0.0777 in Case 1 and ± 0.0918 in Case 2. Thus, the ERD smaller than this limit is judged to be non-zero. In Case 1, no significant ERD was found in any channels until 3 s after the onset of the swallowing. After then, significant ERD was found in the bilateral prefrontal areas (corresponding to the Fp1, Fpz, and Fp2) and the right premotor area (corresponding to the F4 channel) 3–4 s after and in those areas (corresponding to the Fp1, Fp2, and F4 channels) 4–5 s after the onset of the swallow. In Case 2, significant ERD was found in the right lateral sensorimotor area (corresponding to the CP6 and FC6), and the right premotor area (corresponding to the F4 channel) and the bilateral temporal areas (T7 and T8 channels) at the beginning of the swallow but was absent from 1 to 5 sec after the onset of swallowing. The 95% confidence limit for the CMC were 0.0249 in Case 1 and 0.0335 in Case 2. Thus, the CMC larger than this limit is judged to be non-zero. However, as CMC was lower than the limit in each channel in both cases, significant CMC was not found during volitional swallowing.

## Discussion

We found the significant ERD in other cortical areas than medial sensorimotor cortices, the individual timing of ERD emergence and no significant CMC during the volitional swallow in two ALS patients with dysphagia showing a prolonged swallow duration.

In Case 1, we found ERD in the prefrontal areas around the time when the EMG activity reached the peak, which mostly corresponded to the pharyngeal phase (Nederkoorn et al., 1999; Vaiman, 2007). The prefrontal cortex is related to higher motor function, such as inhibition of reaction, control of voluntary movements, and cognitive functions such as attention and working memory (Faw, 2003). In previous studies, ERDs were observed in the dorsolateral prefrontal cortex during self-paced movements, reflecting cognitive processes supporting movement tasks such as motor memory, estimation of time interval for motor preparation, and attention (Rektor et al., 2006; Sochurková et al., 2006). Therefore, the ERD in the prefrontal areas in Case 1 suggests that swallowing was forced with cognitive efforts to compensate for reduced motor outputs due to the possible degeneration of the cortico-bulbar tracts and the lower cranial motor neurons. In Case 2, we found the ERD in the right lateral sensorimotor areas most likely corresponding to the tongue representation. This suggests that activation may be related to weakened tongue movements during the oral phase. In a previous EEG study, ERD was induced in the lateral sensorimotor areas when the tongue was thrusting (Sakihara and Inagaki, 2015). Activation has also been reported in more medial sensorimotor areas during volitional swallowing in healthy volunteers (Hamdy et al., 1996; Dziewas et al., 2009). ERD in the more lateral areas during volitional swallow in Case 2 suggests that activation in the tongue-related areas may reflect an increase in motor outputs to the weakened tongue muscles to transfer into the pharynx as well as compensation for reduced activity of the M1 pharyngeal areas located more medially. Furthermore, significant ERD were found in the bilateral temporal areas in Case 2. Previous reports showed the temporal lesion produced dysphagia and the brain activity was increased in the dysphagic patients (Hamdy et al., 1998; Li et al., 2009b). The detailed mechanism of temporal activities has not been clarified. However, it may be related to the compensation for the swallowing dysfunction.

Although there was almost no difference of the ALS severity according to the scores of ALSFRS-R between Case 1 and 2, Case 1 showed hoarseness due to incomplete vocal closure caused by vocal atrophy. It suggests that Case 1 required more efforts to compensate the incomplete closure of the vocal cord and to prevent aspiration during the swallowing than Case 2, who showed no dysfunction of vocal cord.

As for laterality, the previous MEG study found significant ERD lateralization to the left hemisphere in healthy subjects and to the right hemisphere in ALS patients with both moderate and severe dysphagia (Teismann et al., 2011). In this case report, the both cases showed the lateralization to the right hemisphere as well as the previous report.

Previous MEG studies analyzed ERD just for one second during swallowing (Teismann et al., 2011). However, patients with dysphagia often show a duration of more than several seconds during swallowing. The timing of the ERD appearance differed between Cases 1 and 2. It might be related to the more severely affected period, for example, the period before or after the EMG peak. Further investigation is necessary in more patients with ALS. The current EEG method to evaluate ERD during the swallow may be used in the assessment of effortfulness of the swallow and in determining the therapeutic target phase in swallow rehabilitation. Our finding may extend the knowledge about the mechanism and alteration of cortical involvement in the swallow in dysphagic ALS.

Recently, ERD has been used to restore motor function in the BMI techniques in patients with neurological diseases (Ramos-Murguialday et al., 2013; Ono et al., 2014). In this case report, we clarified the difference of time-course of ERD emergence in the individual ALS patients. If ERD during the swallow can be used in the swallow rehabilitation, the target timing of the ERD needs to be considered. Our findings would be useful in new swallow rehabilitation using ERD. Furthermore, a simpler method using EEG with a fewer electrodes and online analysis of ERD would be necessary for clinical application in future.

We did not find a significant CMC in either case. CMC can measure the cortical control of peripheral motor neurons (Mima and Hallett, 1999a,b). A previous study reported that CMC in ALS patients was significantly diminished relative to healthy subjects during a light hand grip (Proudfoot et al., 2018). The CMC was not significant during volitional swallowing in this study, probably due to cortico-bulbar dysfunction in ALS.

In this study, we characterized the time course and the distribution of ERD during prolonged volitional swallowing in two ALS patients with dysphagia. Although they showed severe dysphagia, which caused difficulties in oral intake, the time course and distribution were different. This suggests that different processes occur for degeneration and compensation in patients with dysphagic ALS. Further studies are necessary in a larger number of patients with ALS.

## Patient Perspective

Case 1 stated that he hoped his result would be helpful for understanding of the impairments of brain activation related to dysphagia in ALS. Case 2 stated that she hoped she could contribute to developing a new therapy to improve dysphagia in ALS by participating in the EEG evaluation.

## Data Availability Statement

The raw data supporting the conclusions of this article will be made available by the authors, without undue reservation.

## Ethics Statement

The studies involving human participants were reviewed and approved by the Committee of Medical Ethics of Dokkyo Medical University. The patients/participants provided their written informed consent to participate in this study.

## Author Contributions

SK designed the study and collected and interpreted the data. AO contributed to data analysis and wrote the initial draft of the manuscript. TT, YT, HI, MM, TMim, TMiz, and KK have contributed to data collection and interpretation and critically reviewed the manuscript. All authors approved the final version of the manuscript.

## Funding

This work was supported by a Grant-in-Aid for Exploratory Research (20K21770), Grants-in-Aid for Scientific Research (B) (21H03308) (SK), Grants-in-Aid for Scientific Research (A) (19H01091) (TMim), Grants-in-Aid for Scientific Research (A) (19H01126), and (B) (19H03939) (KK) from the Japan Society for the Promotion of Science.

## Conflict of Interest

The authors declare that the research was conducted in the absence of any commercial or financial relationships that could be construed as a potential conflict of interest.

## Publisher's Note

All claims expressed in this article are solely those of the authors and do not necessarily represent those of their affiliated organizations, or those of the publisher, the editors and the reviewers. Any product that may be evaluated in this article, or claim that may be made by its manufacturer, is not guaranteed or endorsed by the publisher.
