# Supplementary material for: Case Report: Event-Related Desynchronization Observed During Volitional Swallow by Electroencephalography Recordings in ALS Patients With Dysphagia
Source: Front Behav Neurosci. 2022 Feb 16;16:798375. doi: 10.3389/fnbeh.2022.798375 (PMC8888887; doi:10.3389/fnbeh.2022.798375)
Supplement: Supplementary file 1 [file Data_Sheet_1.pdf]

CARE case report guidelines

CARE Checklist of information to include when writing a case report

(cc) BY-NC-ND

Topic	Item	Checklist item description	Reported on Line
Title	1	The diagnosis or intervention of primary focus followed by the words "case report"	1
Key Words	2	2 to 5 key words that identify diagnoses or interventions in this case report, including "case report"	118-119
Abstract (no references)	3a	Introduction: What is unique about this case and what does it add to the scientific literature?	L9-L13 in the section of abstract
	3b	Main symptoms and/or important clinical findings	L14-L15, L18-L19 in the section of abstract
	3c	The main diagnoses, therapeutic interventions, and outcomes	L19-L25 in the section of abstract
	3d	Conclusion—What is the main "take-away" lesson(s) from this case?	L25-L27 in the section of abstract
Introduction	4	One or two paragraphs summarizing why this case is unique (**may include references**)	L1-L44 in the section of introduction
Patient Information	5a	De-identified patient specific information.	L1, L14 in the section of cases
	5b	Primary concerns and symptoms of the patient.	L1, L14 in the section of cases
	5c	Medical, family, and psycho-social history including relevant genetic information	L1, L14 in the section of cases
	5d	Relevant past interventions with outcomes	L5-7, L16-18 in the section of cases
Clinical Findings	6	Describe significant physical examination (PE) and important clinical findings.	L1- L24 in the section of cases
Timeline	7	Historical and current information from this episode of care organized as a timeline	L1- L24 in the section of cases
Diagnostic Assessment	8a	Diagnostic testing (such as PE, laboratory testing, imaging, surveys). L55 in the section of discussion	L12, L20 in the section of cases
	8b	Diagnostic challenges (such as access to testing, financial, or cultural)	L12, L20 in the section of cases
	8c	Diagnosis (including other diagnoses considered)	L13, L25 in the section of cases
	8d	Prognosis (such as staging in oncology) where applicable	L14, L26 in the section of cases
Therapeutic Intervention	9a	Types of therapeutic intervention (such as pharmacologic, surgical, preventive, self-care)	L1-L31 in the section of EEG and EMG Recordings
	9b	Administration of therapeutic intervention (such as dosage, strength, duration)	L1-L31 in the section of EEG and EMG Recordings
	9c	Changes in therapeutic intervention (with rationale)	L1-L31 in the section of EEG and EMG Recordings
Follow-up and Outcomes	10a	Clinician and patient-assessed outcomes (if available)	—
	10b	Important follow-up diagnostic and other test results	L1-L2 in the section of assessment
	10c	Intervention adherence and tolerability (How was this assessed?)	L3-L4 in the section of assessment
	10d	Adverse and unanticipated events	L6-L7 in the section of assessment
Discussion	11a	A scientific discussion of the strengths AND limitations associated with this case report	L5-L40 in the section of discussion
	11b	Discussion of the relevant medical literature **with references**.	L5-L40 in the section of discussion
	11c	The scientific rationale for any conclusions (including assessment of possible causes)	L55 in the section of discussion
	11d	The primary "take-away" lessons of this case report (without references) in a one paragraph conclusion	L85 in the section of discussion
Patient Perspective	12	The patient should share their perspective in one to two paragraphs on the treatment(s) they received	L1 in the section of patient perspective
Informed Consent	13	Did the patient give informed consent? Please provide if requested	Yes ☑ No ☐
